# Tree-ring δ¹³C and growth responses of *Larix principis-rupprechtii* to climate change in the Central-Northern Taihang Mountains

**DOI:** 10.3389/fpls.2026.1896087

**Published:** 2026-08-13

**Authors:** Jianqiang Dong, Huifang Zhang, Jing Sun, Changming Ma, Yanchao Wang, Chunyou Li

**Affiliations:** 1 Department of Forest Cultivation, College of Forest, Hebei Agricultural University, Baoding, China; 2 College of Mathematics and Information Technology, Xingtai University, Xingtai, China; 3 College of Chemical Engineering and Biotechnology, Xingtai University, Xingtai, China; 4 Hebei Key Laboratory of Digital Freshwater Aquaculture Technology, Xingtai University, Xingtai, China

**Keywords:** basal area increment, intrinsic water-use efficiency, *Larix principis-rupprechtii*, Taihang Mountains, tree-ring δ13C

## Abstract

In the context of global warming and aridification, the Taihang Mountains, recognized as a climate-sensitive transitional zone, necessitate a thorough investigation into the adaptive mechanisms of their forest ecosystems. This study investigates the 60-year variations and climate responses of tree-ring stable carbon isotopes (δ¹³C), intrinsic water-use efficiency (iWUE), and basal area increment (BAI) in *Larix principis-rupprechtii* from the central-northern Taihang Mountains. The results reveal a phased trend in tree-ring δ¹³C, with an increase from 1964 to 2012 followed by a subsequent decline, suggesting dynamic regulation of the ratio of intercellular to atmospheric CO_2_ concentrations (C_i_/C_a_) that may be associated with changes in moisture availability after 2012. iWUE increased significantly during the study period, primarily driven by the combined effects of rising atmospheric CO_2_ concentrations and stomatal regulation. Carbon isotope discrimination (Δ¹³C) exhibited pronounced seasonal responses to climatic factors. Specifically, water availability was the primary determinant of isotopic fractionation during the April–August growing season, while temperature further intensified water stress by increasing evaporative demand. iWUE demonstrated greater sensitivity to climatic variation than BAI. Although their long-term trends showed general consistency, the interannual coupling remained weak. Commonality analysis indicated that C_a_ and relative humidity explained 89.62% of iWUE variation, whereas iWUE and relative humidity explained only 20.55% of BAI variation, with the independent contribution of iWUE being minimal. These results suggest that persistent water stress limits the effective conversion of increased iWUE into radial growth, potentially counteracting the effects of CO_2_ fertilization, and that increases in iWUE do not consistently promote tree growth. This study offers essential insights into the future carbon sequestration potential of central-northern Taihang Mountain forests and adaptive management.

## 1 Introduction

Under accelerating global climate change, rising atmospheric CO_2_ concentrations are contributing to substantial warming and changes in regional hydrological regimes, with profound effects on the structure and function of terrestrial ecosystems (Yuan et al., 2025). Forest ecosystems, as a major component of the terrestrial carbon cycle, play a crucial role in carbon sequestration and climate regulation (Anderegg et al., 2020; Besnard et al., 2025). Throughout their long-term growth, trees adapt to environmental changes by regulating stomatal behavior, photosynthetic processes, and water-use strategies, which are systematically recorded in tree-ring structures and stable isotope compositions (Saurer et al., 2004; Liu et al., 2014a; Huang et al., 2024).

Tree-ring stable carbon isotopes (δ¹³C) provide valuable insights into photosynthetic rates, stomatal conductance, and environmental water conditions, making them widely used indicators in climate reconstructions and tree physiological ecology research (Gessler et al., 2014; Lu et al., 2024; Liu et al., 2014b). Compared with conventional tree-ring width metrics, δ¹³C can provide a more direct record of physiological responses to environmental changes, particularly during drought and water-limited conditions. In C_3_ plants, the δ¹³C of tree-ring cellulose is predominantly influenced by the ratio of intercellular CO_2_ concentration (C_i_) to atmospheric CO_2_ concentration (C_a_), which reflects the combined effects of temperature, water availability, and atmospheric CO_2_ levels (Farquhar et al., 1982; McCarroll and Loader, 2004). Water availability affects C_i_ by regulating stomatal opening, whereas temperature primarily influences carbon isotope fractionation indirectly by modifying photosynthetic enzyme activity and atmospheric evaporative demand (Kelly et al., 2016). The calculation of carbon isotope discrimination (Δ¹³C) accounts for changes in atmospheric δ¹³C since the Industrial Revolution, thereby enabling a more accurate assessment of climatic influences on tree physiological processes (Leonardi et al., 2012).

In recent years, intrinsic water-use efficiency (iWUE) derived from tree-ring δ¹³C has become a significant proxy for assessing forest responses to rising atmospheric CO_2_ concentrations and climate change (Battipaglia et al., 2013; Rahman et al., 2020). Numerous studies indicate that while iWUE generally increases across various regions and species under elevated CO_2_, the extent to which this increase promotes radial growth remains unclear (Penuelas et al., 2011; Wang et al., 2021; Zhang et al., 2026). Increases in iWUE may promote tree growth by enhancing photosynthetic carbon assimilation or by reducing water use per unit of carbon fixed. Conversely, under warming conditions, enhanced evapotranspiration combined with intensified water stress can lead to prolonged stomatal closure, which restricts carbon uptake and results in limited or negligible radial-growth enhancement (Heilman et al., 2021). Therefore, integrating iWUE with radial growth metrics, such as basal area increment (BAI), aids in disentangling CO_2_ fertilization effects from climatic stress, thereby elucidating the coupling mechanisms between physiological regulation and growth responses. This approach is essential for a comprehensive understanding of tree adaptive responses to climate change and their potential for carbon sequestration.

The Taihang Mountains, located within a critical transitional zone influenced by the East Asian monsoon, form an important geographical transition between the North China Plain to the Loess Plateau and demonstrate significant sensitivity to regional climatic fluctuations. Recent observations indicate a significant warming trend in the region, accompanied by heightened variability in annual precipitation and decreased relative humidity, conditions that may increase water stress and affect montane coniferous forest ecosystems (Hou et al., 2024). *Larix principis-rupprechtii*, the dominant species in mid- to high-altitude regions of the Taihang Mountains, shows considerable sensitivity to fluctuations in water availability and temperature, making it a suitable species for investigating the responses of montane forest ecosystems to climate change (Tian et al., 2025). However, existing studies have primarily concentrated on tree-ring width or climate reconstruction (Cai and Liu, 2013; Zhang et al., 2017; Wang et al., 2024), while comprehensive studies that examine tree-ring stable carbon isotopes, physiological regulation, and their relationship with radial growth in *Larix principis-rupprechtii* in the Taihang Mountains are limited. Specifically, there is an inadequate understanding of the dynamics of iWUE and their driving mechanisms across interannual to decadal scales. This knowledge gap has constrained a comprehensive understanding of the adaptive capacity and carbon–water coupling processes within Taihang montane forest ecosystems. The present study addresses this gap by examining the long-term physiological and growth responses of these mountain forests to climate change.

The present study focuses on *Larix principis-rupprechtii* in the central-northern Taihang Mountains, systematically analyzing nearly 60 years of tree-ring δ¹³C, Δ¹³C, iWUE, and BAI. The study aims to investigate: (1) the long-term trends of tree-ring stable carbon isotopes and iWUE in *Larix principis-rupprechtii* within the Taihang Mountains; (2) the response patterns of tree-ring carbon isotopes to climatic factors and the identification of their primary controlling factors; and (3) the quantification of the differential sensitivity of iWUE and radial growth to climate change, as well as the partitioning of the independent and combined contributions of atmospheric CO_2_ and climatic factors to variations in iWUE and tree growth. The findings provide a scientific basis for understanding the adaptive mechanisms of Taihang Mountain forest ecosystems in response to climate change and for predicting their future trajectories.

## 2 Materials and methods

### 2.1 Study site

**Figure 1:**
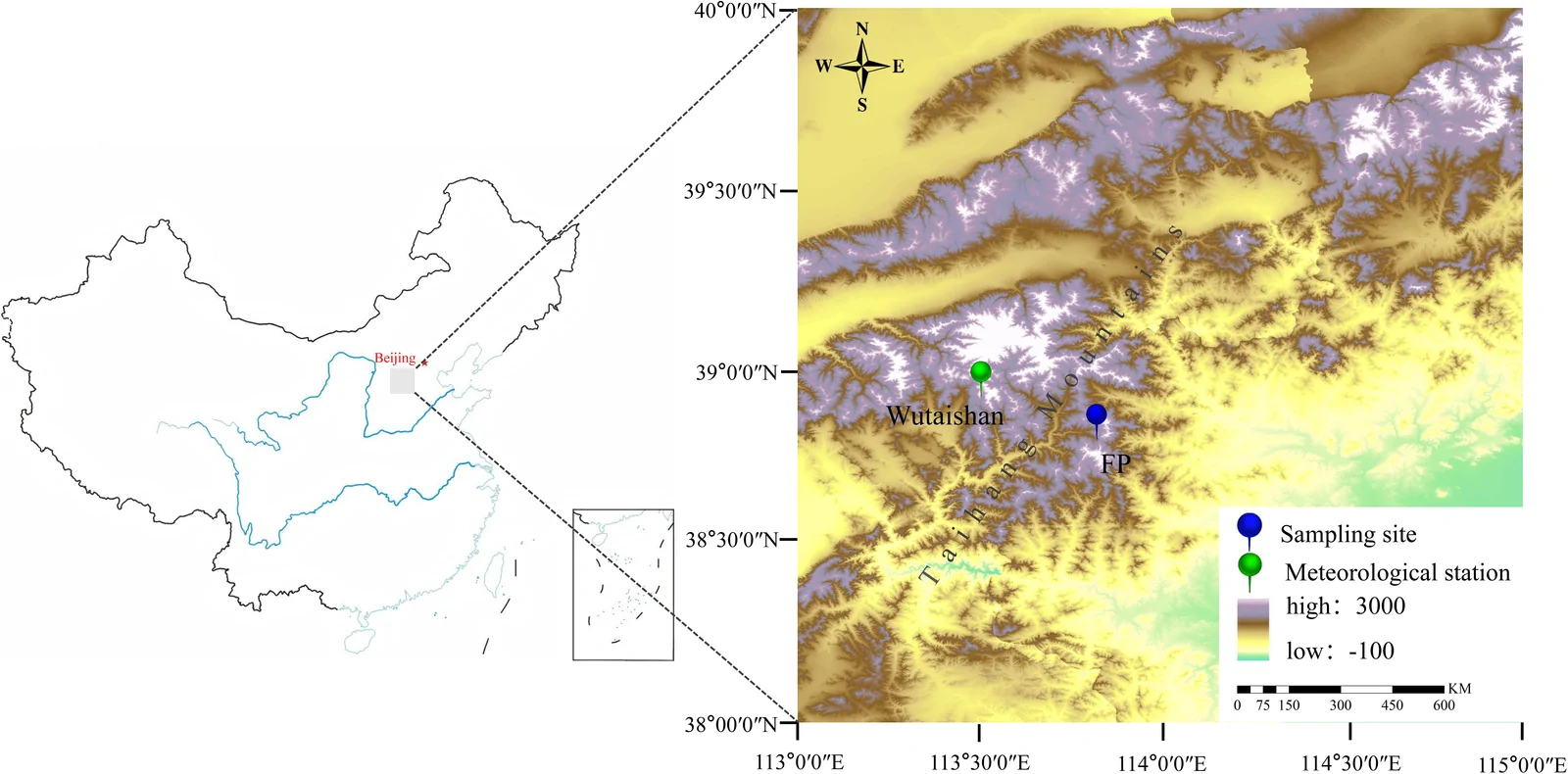
Study area, distribution of sampling site, and locations of nearby meteorological stations.

The study area is located in Fuping (FP) on the eastern slope of the central–northern Taihang Mountains (113°50′E, 38°50′N; 2088 m a. s. l) (**Figure 1**). It is characterized by complex mountainous terrain, steep slopes, pronounced relief, and substantial local environmental heterogeneity. The bedrock is primarily composed of granite and gneiss, with mountain brown soils and brown earth as the dominant soil types. Soils are generally shallow and exhibit considerable spatial variation in thickness. The dominant tree species in the study area is *Larix principis-rupprechtii*, which primarily occurs at middle to high elevations and often forms pure stands. The understory vegetation is diverse, primarily comprising shrubs and herbaceous species. Common shrub species include *Lonicera* spp. and *Lespedeza* spp., whereas the herbaceous layer is dominated by Poaceae and Asteraceae species. The East Asian summer monsoon significantly influences the moisture conditions in the area, characterized by the northward progression of warm and humid air masses from the western Pacific and Indian Oceans under monsoonal circulation during the summer. The topography of the Taihang Mountains induces orographic blocking and uplift, leading to moisture convergence on the windward slopes. This phenomenon significantly influences vegetation growth and the regional hydrological cycle. In contrast, winter conditions are dominated by the Siberian High, which brings prevailing cold and dry air masses that restrict moisture input. Consequently, the region experiences a pronounced seasonal contrast in water availability.

### 2.2 Sample collection and BAI calculation

A total of 24 healthy *Larix principis-rupprechtii* trees without visible signs of pest damage or human disturbance were selected for sampling within the study area. Two cores were extracted from each tree at breast height (1.3 m) along mutually perpendicular directions, resulting in a total of 48 cores. The samples were carefully processed and cross-dated following established dendrochronological protocols (Cook and Kairiukstis, 1990; Stokes and Smiley, 1996). Annual ring widths were measured using a binocular microscope and a LINTAB measuring system, achieving a precision of 0.01 mm. The accuracy of cross-dating was verified using the COFECHA program (Holmes, 1983). The tree-ring width series were standardized using the ARSTAN program to remove age- and size-related growth trends. The standardized series were then combined using a robust mean to construct the tree-ring width index chronology (Cook and Kairiukstis, 1990). The resulting chronology and annual sample depth are presented in **Supplementary Figure 1**. Although the tree-ring width index chronology effectively reflects interannual growth variability, the standardization procedure may part of the low-frequency signal. Therefore, BAI was used as the primary growth indicator because it reduces the geometric effect associated with increasing stem size while retaining long-term growth variability (Penuelas et al., 2008), and was calculated using Equation 1:

(1) $$BAI=\pi \times (R_{n}^{2}-R_{n-1}^{2})$$

where BAI denotes the basal area increment (cm² year^-^¹), and *R_n_* and *R_n−1_* represent the tree radii at breast height for years n and n−1, respectively. The BAI chronology covers the period from 1918 to 2023.

### 2.3 Analysis of tree-ring stable carbon isotopes

Four representative trees were selected, and one core from each tree was used for isotope analysis, based on the following criteria: (1) the absence of visible damage or abnormal growth; (2) a high correlation between the selected core and the master chronology; and (3) clearly distinguishable annual ring boundaries. These selection criteria were intended to improve the representativeness of the resulting δ¹³C chronology for the study site. To mitigate juvenile effects on tree-ring isotopes (Leavitt and Long, 1984; Hemming et al., 1998; Szymczak et al., 2012), the first 30 years of each core were excluded from the analysis. Under carbon-free conditions, each annual ring was meticulously separated under a microscope using fine, sharp blades. Detailed procedures for α-cellulose extraction are outlined in the reference (Liu et al., 2018). The extracted cellulose was then homogenized using an ultrasonic cell disrupter. Approximately 110–140 μg of homogenized α-cellulose was weighed and encapsulated in tin capsules for subsequent analysis. Carbon was oxidized to CO_2_ using a FLASH 2000 elemental analyzer coupled with a dynamic combustion system. The resulting CO_2_ was then introduced into a DELTA V Advantage isotope ratio mass spectrometer to measure the ¹³C/¹²C ratio. A standard cellulose sample (IAEA CH3, δ¹³C = –24.724‰) was included after every seven samples, resulting in a measurement precision of 0.18‰. δ¹³C values were referenced to the Vienna Pee Dee Belemnite (VPDB) standard (Coplen, 1995). The carbon isotope ratio of tree-ring cellulose was expressed as δ¹³C and calculated using Equation 2:

(2) $$\delta^{13}C=(R_{sample}/R_{s\tan dard}-1)\times 1000$$

where *R_sample_* and *R_standard_* represent the ¹³C/¹²C ratios of the sample and the standard material, respectively. For each individual tree core, α-cellulose was chemically extracted and analyzed using isotope ratio mass spectrometry (IRMS). The mean δ¹³C values were subsequently calculated employing the Numerical Mixing Method (NMM) (Liu et al., 2012) to construct a single stable isotope chronology. The chronology spans the period from 1964 to 2023.

The Δ¹³C was calculated to account for the long-term decline in atmospheric δ¹³C associated with increasing atmospheric CO_2_ derived from fossil-fuel combustion since the Industrial Revolution (De Jong et al., 1979). Data on atmospheric CO_2_ concentrations required for this calculation were obtained from the Mauna Loa Observatory (https://scrippsco2.ucsd.edu/data/atmospheric-co2-data/sampling-station-records/mauna-loa-observatory-hawaii/), while δ¹³C_a_ values were derived from the global δ¹³C_CO_2_ composite series of the Scripps CO_2_ Program (https://scrippsco2.ucsd.edu/). Carbon isotope discrimination was calculated using Equation 3:

(3) $${\text{Δ}}^{13}\text{C}=\frac{{\text{δ}}^{13}{\text{C}}_{\text{a}}-{\text{δ}}^{13}{\text{C}}_{\text{p}}}{1+{\text{δ}}^{13}{\text{C}}_{\text{p}}/1000}$$

where δ¹³C_a_ and δ¹³C_p_ represent the carbon isotope ratios of atmospheric CO_2_ and tree-ring cellulose, respectively.

For C_3_ plants, Δ¹³C is closely associated with C_i_ and C_a_ (Farquhar et al., 1982), and this relationship is expressed in Equation 4:

(4) $${\text{Δ}}^{13}C=a+(b-a)\times (C_{i}/C_{a})$$

where *a* represents the fractionation constant for CO_2_ diffusion through stomata (4.4‰), *b* indicates the fractionation constant associated with carboxylation (27‰), and C_i_ and C_a_ signify the intercellular and atmospheric CO_2_ concentrations, respectively.

iWUE is defined as the ratio of the photosynthetic assimilation rate (A) to stomatal conductance for water vapor (g_s_). Based on the mechanistic model relating Δ¹³C to the C_i_/C_a_ ratio, iWUE was calculated using Equation 5 (Ehleringer and Dawson, 1992):

(5) $$\text{iWUE}=A/g_{s}=C_{a}\times [(1-C_{i}/C_{a})/1.6]$$

Equation 5 can be rearranged to obtain Equation 6:

(6) $$\text{iWUE}=\frac{C_{a}-C_{i}}{1.6}$$

Thus, iWUE can be characterized by both the C_i_/C_a_ ratio and the absolute difference between C_a_ and C_i_. There are three common scenarios describing the relationship between C_i_ and C_a_, representing distinct response patterns of C_i_ to increasing C_a_ (Saurer et al., 2004): (1) C_i_ remains constant; (2) the C_i_/C_a_ ratio remains constant; and (3) the C_a_–C_i_ difference remains constant. Under these scenarios, C_i_ exhibits different patterns of change in response to C_a_: (1) it remains unchanged, (2) it changes proportionally, or (3) it changes at a constant rate. In this study, the determinants of iWUE were assessed by comparing the observed C_i_/C_a_ ratios with theoretical expectations for each scenario.

### 2.4 Meteorological data

**Figure 2:**
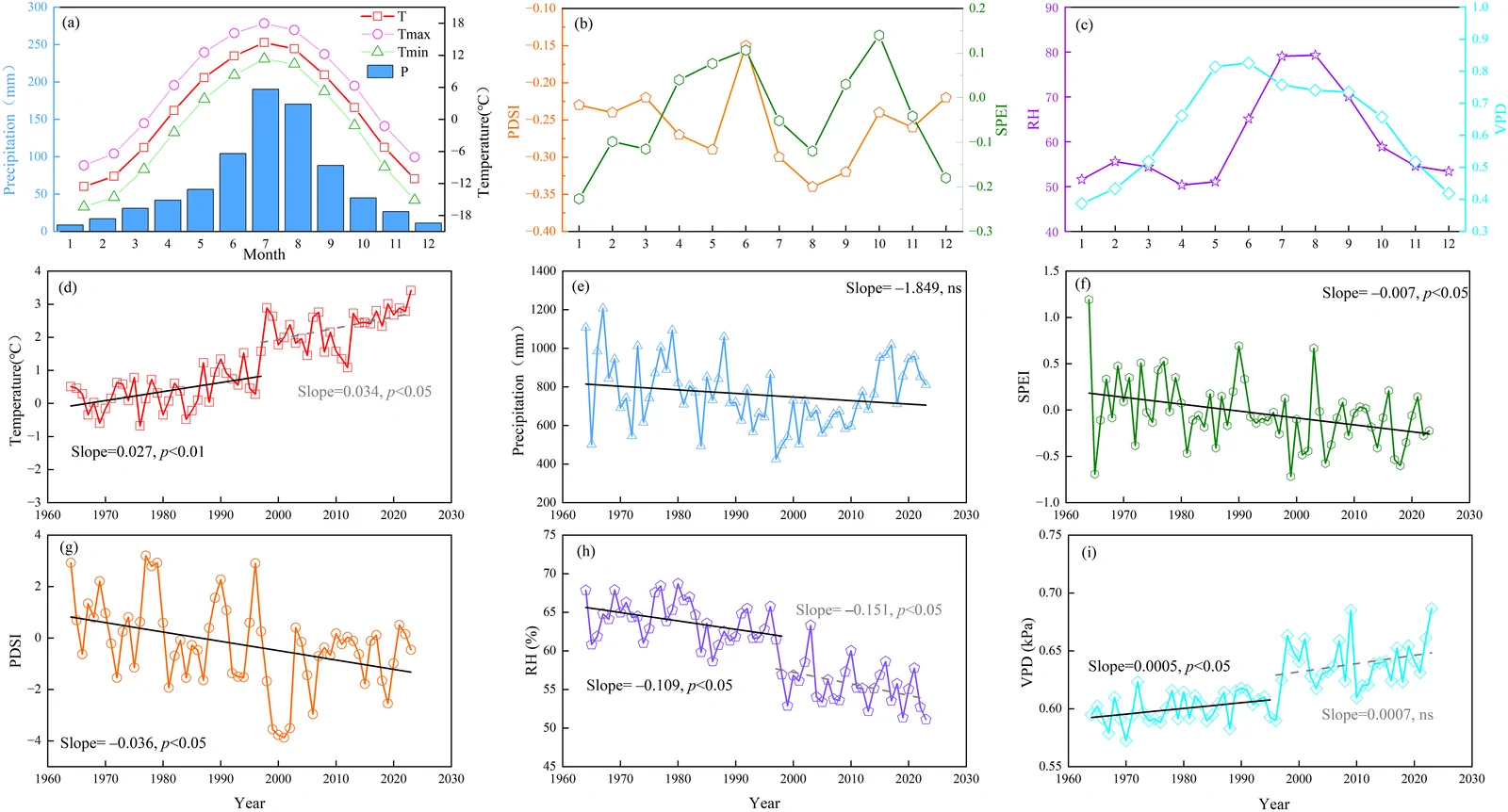
Climate data from the Wutaishan Meteorological Station. **(a)** Monthly mean, maximum, and minimum temperatures, and total monthly precipitation; **(b)** Monthly mean PDSI and SPEI; **(c)** monthly mean RH and VPD; Interannual variations from 1964 to 2023 of **(d)** annual mean temperature, **(e)** annual total precipitation, **(f)** SPEI, **(g)** PDSI, **(h)** annual mean RH, and **(i)** annual mean VPD. ns, no significant trends. Diagonal lines represent climate change trends.

Meteorological data were obtained from the China National Meteorological Information Center (http://data.cma.cn/site/index.html). Data from the Wutaishan Meteorological Station (113°31′E, 38°57′N, 2210 m a. s. l), located near the study area, were used to characterize regional climate variability. The time series of meteorological data spans from 1964 to 2023. The homogeneity of the observed meteorological data was evaluated using the Standard Normal Homogeneity Test (SNHT) (Peterson and Easterling, 1994). No significant inhomogeneity was detected, indicating that the station records were suitable for subsequent analyses. The meteorological variables included monthly and annual precipitation, mean temperature, maximum temperature, minimum temperature, relative humidity (RH), and vapor pressure deficit (VPD) (**Figure 2**). Drought intensity was quantified using the 1-month Standardized Precipitation Evapotranspiration Index (SPEI-1), obtained from the SPEI base global drought index database (https://spei.csic.es/database.html), and the Palmer Drought Severity Index (PDSI), retrieved from the KNMI Climate Explorer (http://climexp.knmi.nl). SPEI-1 was selected because its one-month accumulation period is sensitive to short-term fluctuations in precipitation and evaporative demand, thereby capturing monthly moisture variations associated with seasonal tree physiological responses (Vicente-Serrano et al., 2010). During 1964–2023, the study area exhibited an annual mean temperature of 1.2 °C, with monthly mean temperatures ranging from –12.6 °C to 14.4 °C. Mean annual precipitation amounted to 759.6 mm, fluctuating between 325.5 mm in 1991 and 1206.9 mm in 1967, mainly concentrated in June–August. The annual mean RH was 59.6%, peaking in July and August. The annual mean VPD was 0.62 kPa, with the highest values occurring in June. Inter-annual climate variability in the study area (**Figure 2**) revealed significant increases in annual mean temperature and VPD, whereas annual precipitation decreased nonsignificantly, and RH, SPEI, and PDSI declined significantly over the observed period. These changes indicate intensified atmospheric dryness and regional moisture deficits, which may affect stomatal conductance and thereby influence tree-ring δ¹³C.

### 2.5 Statistical analysis

Potential change points in the annual climate series and tree-ring δ¹³C and Δ¹³C chronologies were identified using the non-parametric Pettitt test. For series with a significant change point (*p* < 0.05), segmented linear regression was performed using the identified change point, whereas ordinary linear regression was applied to series without a significant change point (Pettitt, 1979). Pearson correlation analysis was employed to assess the relationships between Δ¹³C and temperature, precipitation, RH, SPEI, PDSI and VPD. Correlation analyses were performed at both monthly and annual temporal scales. Since tree growth may be influenced by climate conditions before and during the growing season, the monthly analysis incorporated climate variables from September of the previous year to October of the current year. This approach was used to identify the climatic periods most closely associated with tree-ring carbon isotope discrimination. All analyses involving δ¹³C, Δ¹³C, iWUE, climate variables, and BAI were conducted over their common period of 1964–2023. At the annual scale, the responses of iWUE and BAI to major climate variables were assessed, and linear regression and correlation analyses were applied to evaluate their differential sensitivities to climate change. To distinguish long-term trends from interannual variability, both the original and first-differenced series of iWUE and BAI were analyzed to assess their interrelationships across multiple temporal scales. Additionally, to address multicollinearity, commonality analysis was performed in R using the yhat package (Courville and Thompson, 2001; Huang et al., 2017) to quantitatively decompose the independent and shared contributions of C_a_ and RH to variation in iWUE, as well as the contributions of RH and iWUE to BAI variation. This analysis elucidated the relative influences of water conditions and physiological regulatory factors on tree growth.

## 3 Results

### 3.1 Characteristics of tree-ring δ¹³C and Δ¹³C series

**Figure 3:**
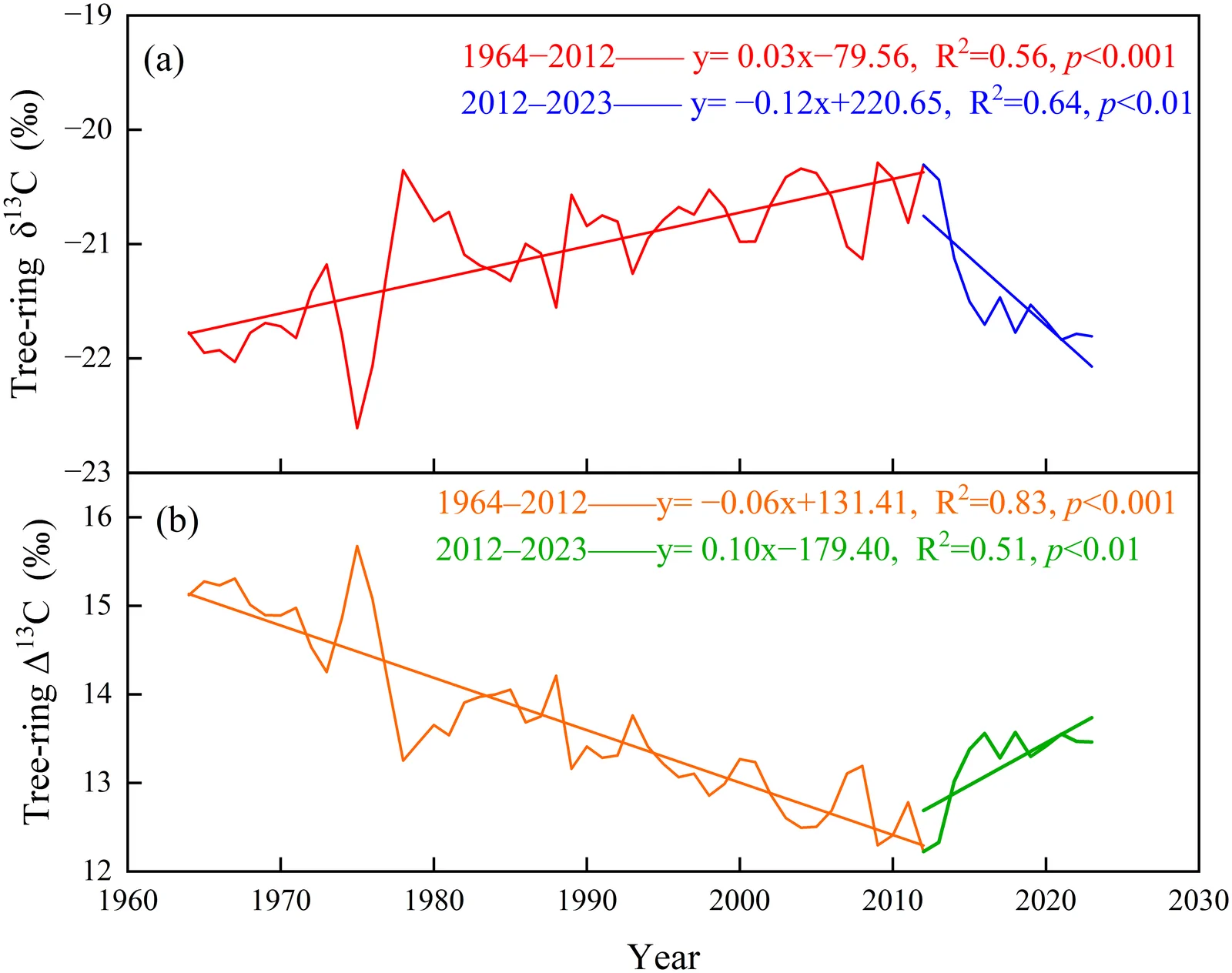
Time series of tree-ring **(a)** δ¹³C and **(b)** Δ¹³C. The colored lines represent the annual observed values, and the superimposed straight lines represent the fitted linear regression trends for 1964–2012 and 2012–2023.

From 1964 to 2023, tree-ring δ¹³C and Δ¹³C of *Larix principis-rupprechtii* displayed significant inter-annual fluctuations and phased long-term variations (**Figure 3**). Across the entire study period, the annual δ¹³C values ranged from –22.61‰ to –20.29‰, with an average of –21.5‰. From 1964 to 2012, δ¹³C showed a sustained upward trend, followed by a marked decline in the past decade. Correspondingly, Δ¹³C values varied from 12.22‰ to 15.67‰, with a mean of 13.64‰. Δ¹³C decreased steadily from the early study period until around 2012, after which it demonstrated a moderate rebound. Overall, δ¹³C and Δ¹³C exhibited a pronounced inverse relationship over the long term.

### 3.2 Trends in C_i_, C_i_/C_a_, and iWUE

**Figure 4:**
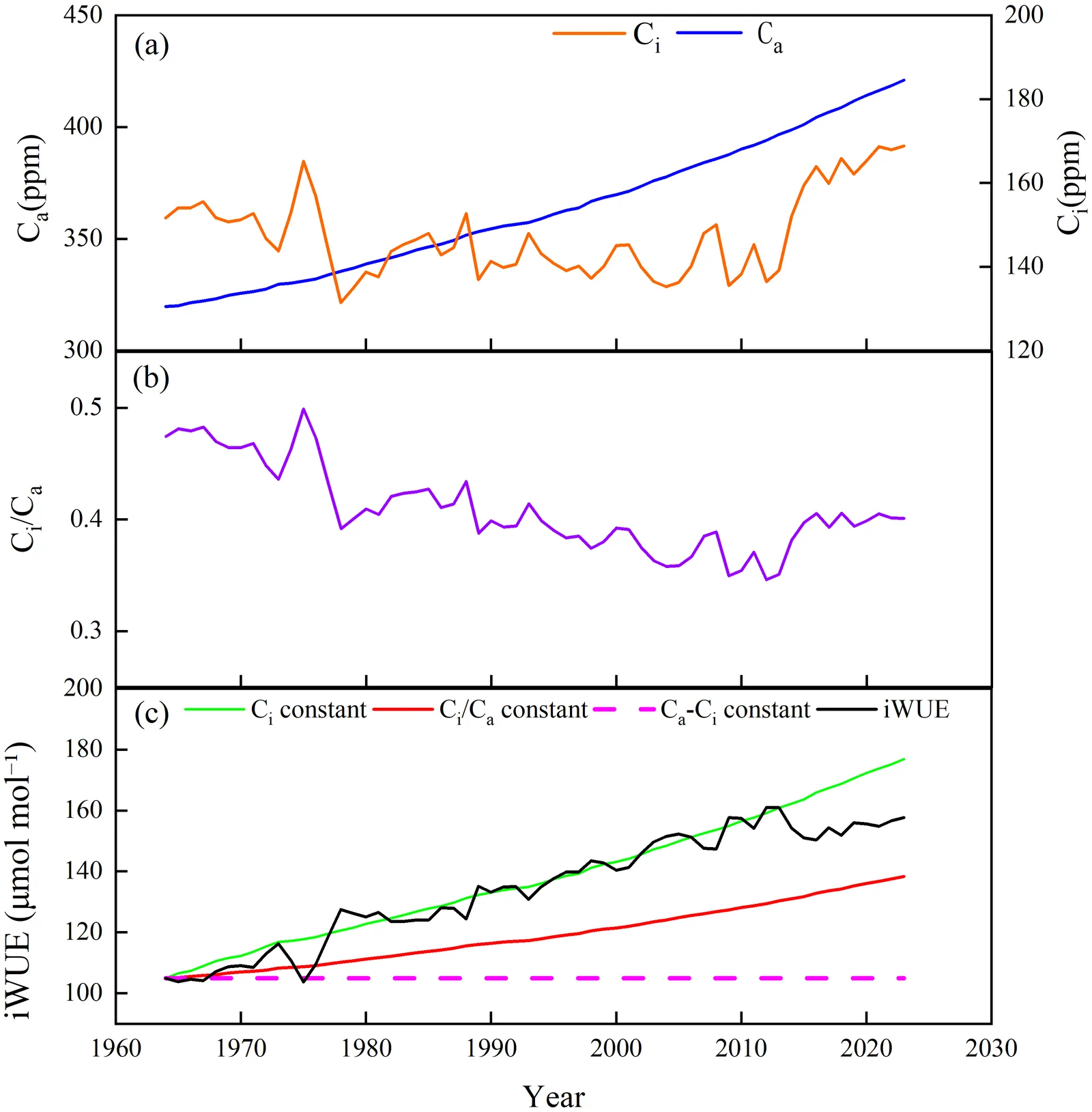
**(a)** Variations of C_i_ and C_a_. **(b)** Changes in the C_i_/C_a_ ratio. **(c)** Calculated iWUE and iWUE derived under three hypothetical scenarios: C_i_ constant, C_i_/C_a_ constant, and C_a_–C_i_ constant.

The long-term variations of C_a_, C_i_, the C_i_/C_a_ ratio, and iWUE from 1964 to 2023 are shown in **Figure 4**. C_a_ exhibited a steady increase over the study period, rising from 319.62 ppm at its onset to 421.08 ppm in recent years, which corresponds to a relative increase of approximately 31.7% and demonstrates a consistent monotonic upward trend. In contrast, C_i_ fluctuated between 131.39 and 168.82 ppm throughout the study period, exhibiting substantial inter-annual variability, with a maximum-to-minimum ratio of approximately 1.29. The C_i_/C_a_ ratio showed an overall declining trend, decreasing from a maximum of 0.48 to a minimum of 0.36, followed by a slight rebound in the later period, yet remaining below its initial value. iWUE showed a marked upward trend, starting from a minimum of 103.75 μmol mol^-^¹ in 1965 and gradually rising to a maximum of 161.02 μmol mol^-^¹ in 2012. From 1964 to 2023, iWUE showed a net increase of 53.91 μmol mol^-^¹, corresponding to an approximate relative increase of 51.96%. Comparisons with various hypothetical scenarios indicate that the observed iWUE was significantly higher than that of the C_a_–C_i_ constant scenario. Around 2012, a turning point occurred, as the relative position shifted from alignment with the C_i_ constant scenario in the early period to an intermediate state between the C_i_ constant and C_i_/C_a_ constant scenarios in the later period.

### 3.3 Correlation of Δ¹³C and climate variables

**Figure 5:**
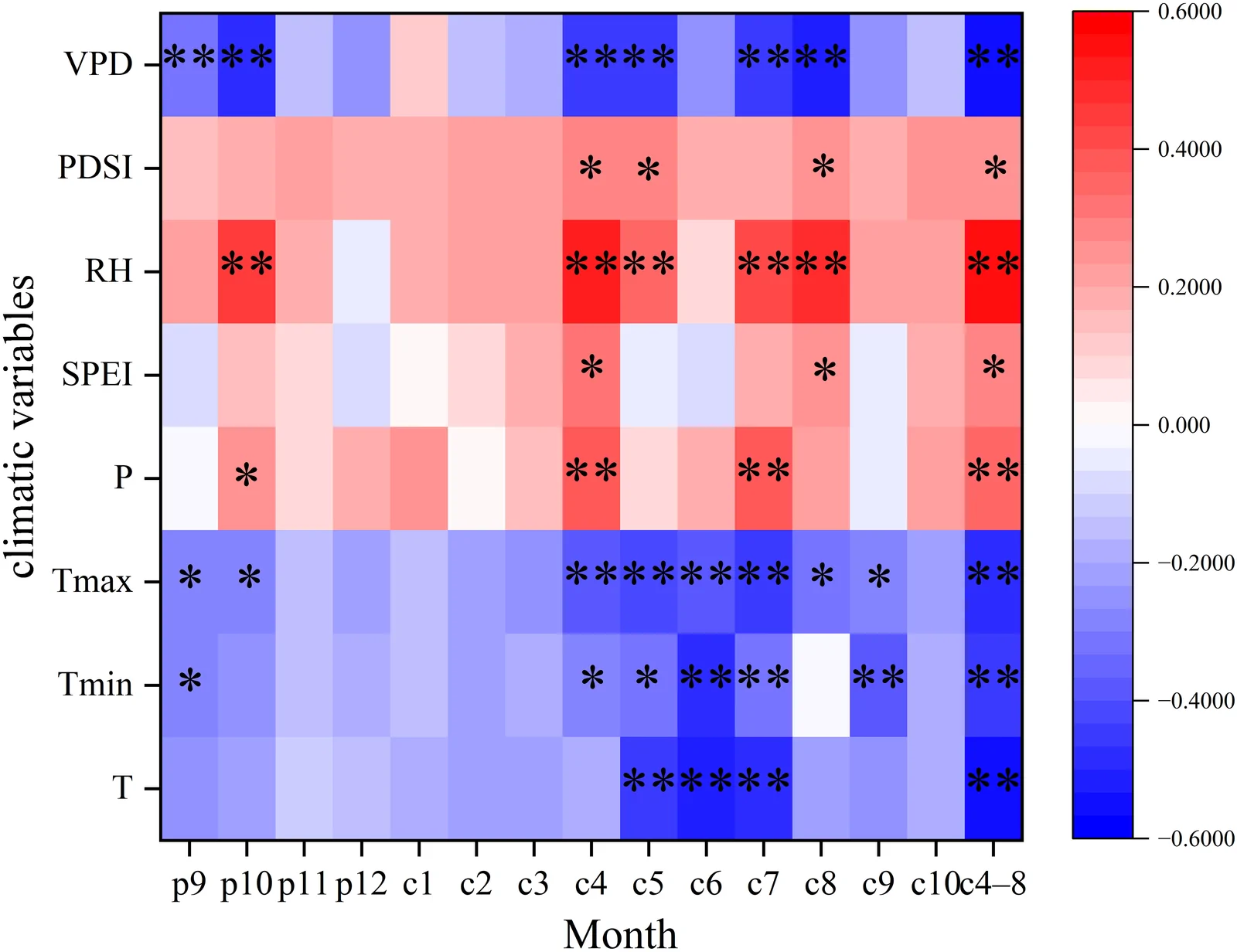
Correlation between the Δ¹³C time series and climatic variables. T, mean temperature; Tmin, mean minimum temperature; Tmax, mean maximum temperature; P, precipitation; SPEI, Standardized Precipitation Evapotranspiration Index; RH, relative humidity; PDSI, Palmer Drought Severity Index; VPD, vapor pressure deficit. p indicates previous year, c indicates current year. *p < 0.05. **p < 0.01.

According to the correlation analysis (**Figure 5**), Δ¹³C of *Larix principis-rupprechtii* displayed marked variation in its relationships with meteorological variables across different months. Overall, Δ¹³C was positively correlated with indicators of moisture availability, including RH, precipitation, SPEI, and PDSI, but negatively correlated with VPD and temperature variables. At the monthly scale, Δ¹³C was significantly positively correlated with RH in October of the previous year and in April, May, July, and August of the current year. Additionally, Δ¹³C exhibited positive correlations with precipitation in October of the previous year and in April and July of the current year, with SPEI in April and August, and with PDSI in April, May, and August of the current year. In contrast, Δ¹³C was significantly negatively correlated with VPD in September and October of the previous year and in April, May, July, and August of the current year. Temperature variables were mostly negatively correlated with Δ¹³C. Specifically, Δ¹³C exhibited significant negative correlations with mean, maximum, and minimum temperatures in May, June, and July of the current year, as well as with maximum and minimum temperatures in September and maximum temperature in October of the previous year. At the single-month scale, the strongest correlation was observed between Δ¹³C and August VPD (r = −0.51, *p* < 0.01). For combined months, Δ¹³C was most strongly correlated with meteorological variables from April to August, all reaching statistical significance (*p* < 0.05). The highest correlation was with VPD (r = −0.56, *p* < 0.01).

### 3.4 Responses of iWUE and BAI to climate variables

**Figure 6:**
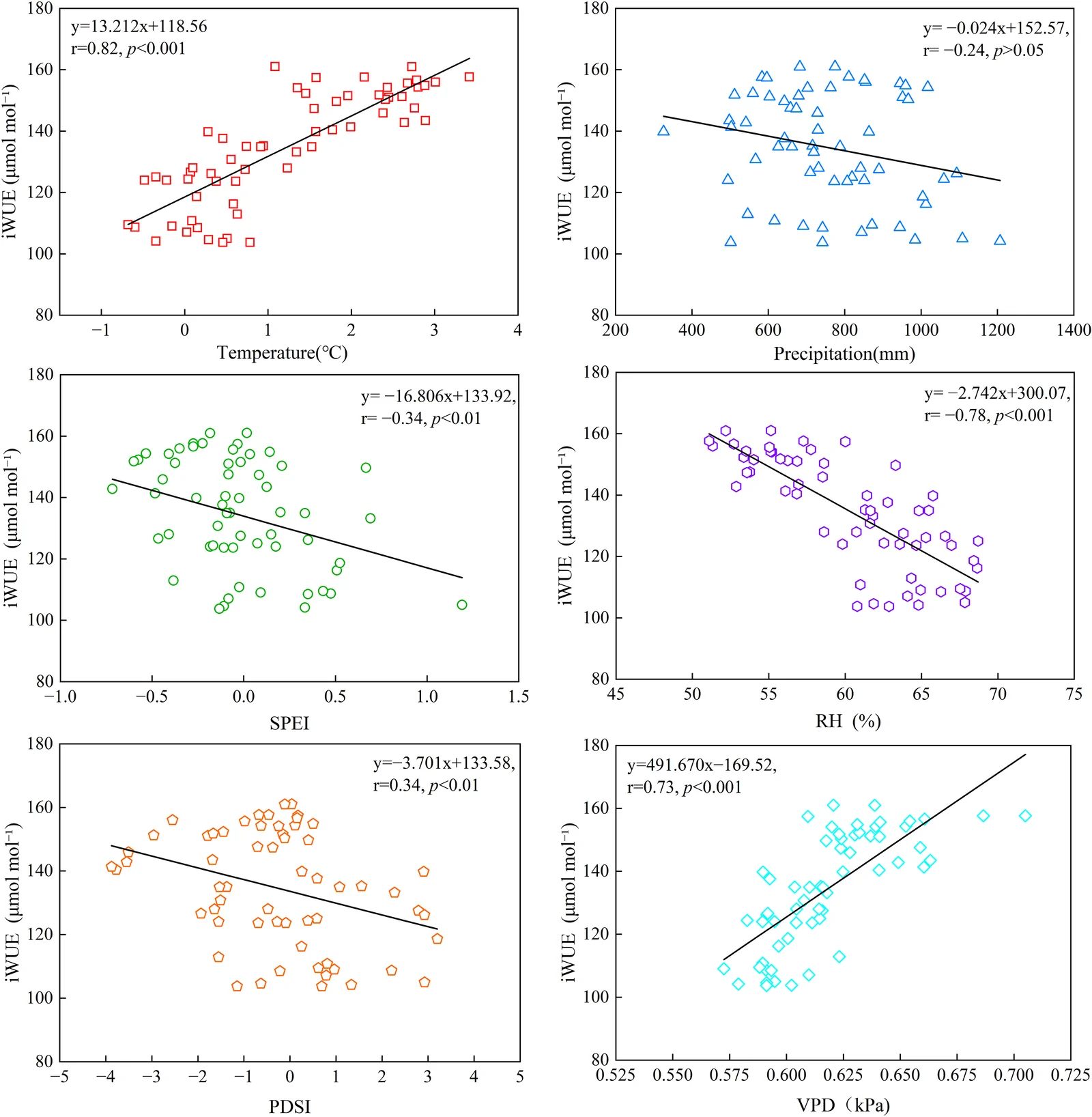
Scatter plots of the relationships between iWUE and annual mean temperature, precipitation, RH, SPEI, PDSI and VPD. The lines represent trends, and the corresponding regression equations and significance levels are also shown.

**Figure 7:**
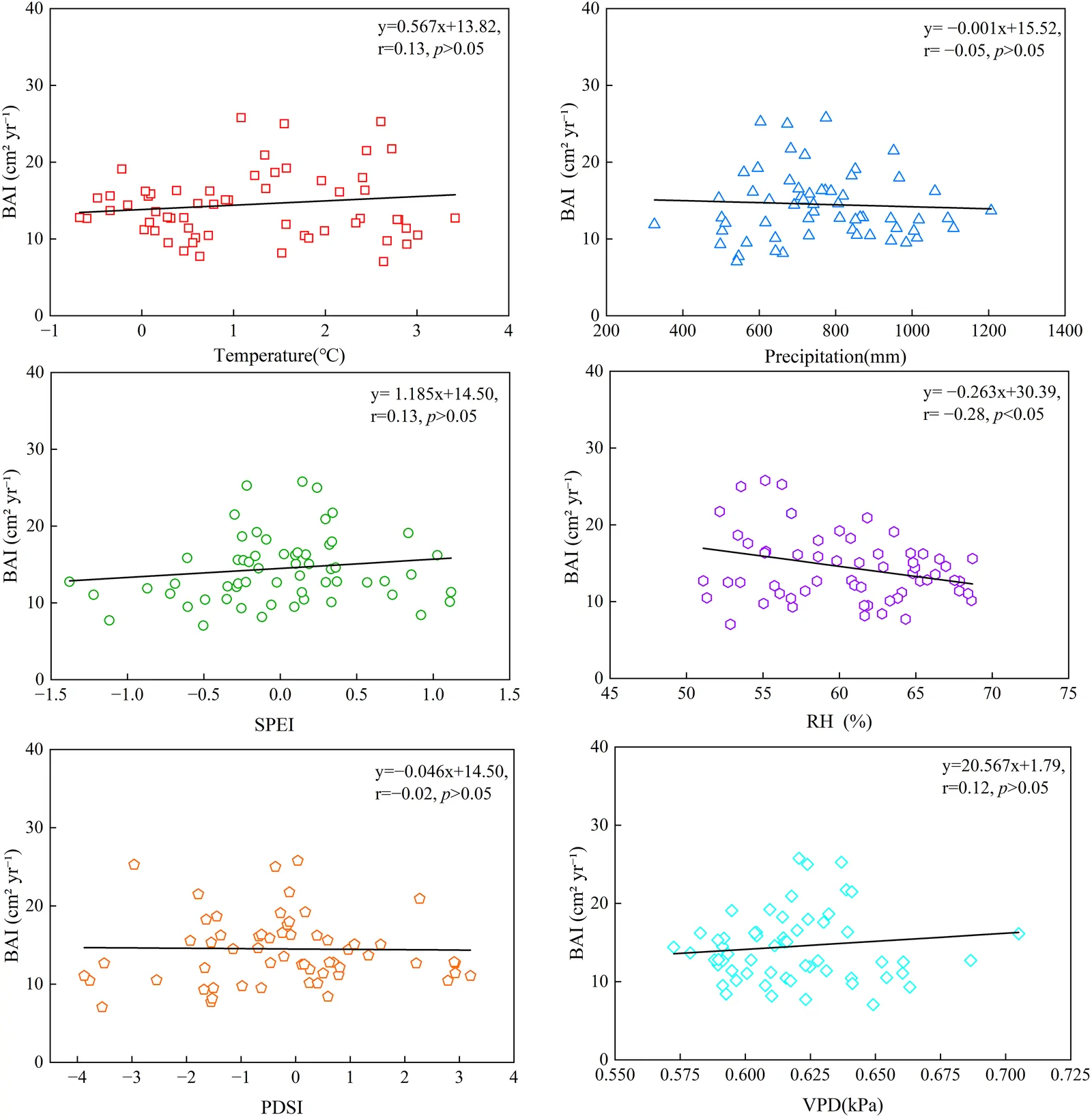
Scatter plots of the relationships between BAI and annual mean temperature, precipitation, RH, SPEI, PDSI and VPD. The lines represent trends, and the corresponding regression equations and significance levels are also shown.

Based on annual-scale climate data, iWUE displayed pronounced responses to major climate variables (**Figure 6**). iWUE was significantly positively correlated with annual mean temperature (r = 0.82, *p* < 0.001) and VPD (r = 0.73, *p* < 0.001), whereas it was significantly negatively correlated with annual SPEI (r = −0.34, *p* < 0.01), PDSI (r = −0.34, *p* < 0.01) and RH (r = −0.78, *p* < 0.001). Additionally, iWUE was negatively correlated with annual mean precipitation (r = −0.24, *p* > 0.05), though the correlation was not statistically significant. In contrast, BAI exhibited a generally weaker direct response to annual-scale climate variables (**Figure 7**). BAI did not show significant correlations with annual mean temperature (r = 0.13, *p* > 0.05), precipitation (r = −0.05, *p* > 0.05), SPEI (r = 0.13, *p* > 0.05), PDSI (r = −0.02, *p* > 0.05), or VPD (r = 0.12, *p* > 0.05), and was significantly negatively correlated only with annual mean RH (r = −0.28, *p* < 0.05). Overall, at the annual scale, iWUE demonstrated a markedly stronger response to climate variability than radial growth, whereas the direct response of radial growth to climate variables was relatively limited.

### 3.5 Relationship between iWUE and BAI

**Figure 8:**
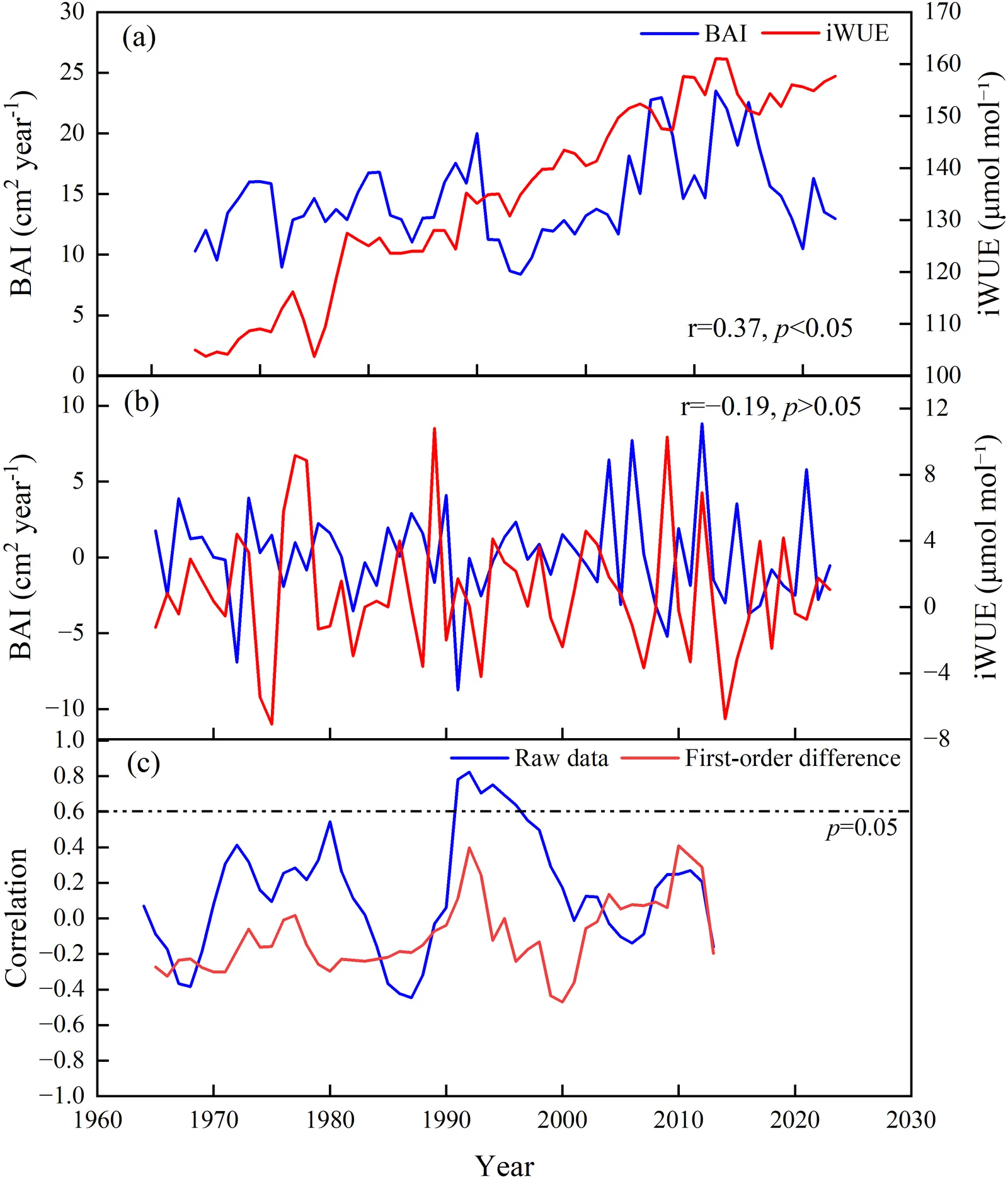
Comparison of iWUE and BAI: **(a)** annual values; **(b)** first-order differenced series; **(c)** 11-year moving correlations. The horizontal dashed lines indicate the 95% confidence level.

**Figure 8** shows the temporal patterns of iWUE and BAI and their interannual relationship. Throughout the study period, iWUE showed a consistent increasing trend. In contrast, BAI exhibited more pronounced interannual fluctuations, primarily ranging between approximately 10 and 25 cm² year^-^¹, with alternating periods of high and low values. Correlation analysis of the original time series indicated a significant positive relationship between iWUE and BAI (r = 0.37, *p* < 0.05) (**Figure 8a**), suggesting moderate synchrony in their long-term trends. However, following the application of first-order differencing to the BAI and iWUE series, the interannual correlation diminished and became non-significant (r = −0.19, *p* > 0.05) (**Figure 8b**), revealing weak interannual coupling. The 11-year moving correlation analysis (**Figure 8c**) demonstrated pronounced temporal non-stationarity, with correlation coefficients of the original series alternating between positive and negative values over time, peaking in 1992 (r = 0.82, *p* < 0.05). Conversely, the moving correlation coefficients of the differenced series ranged from −0.47 to 0.41 and did not achieve statistical significance.

**Figure 9:**
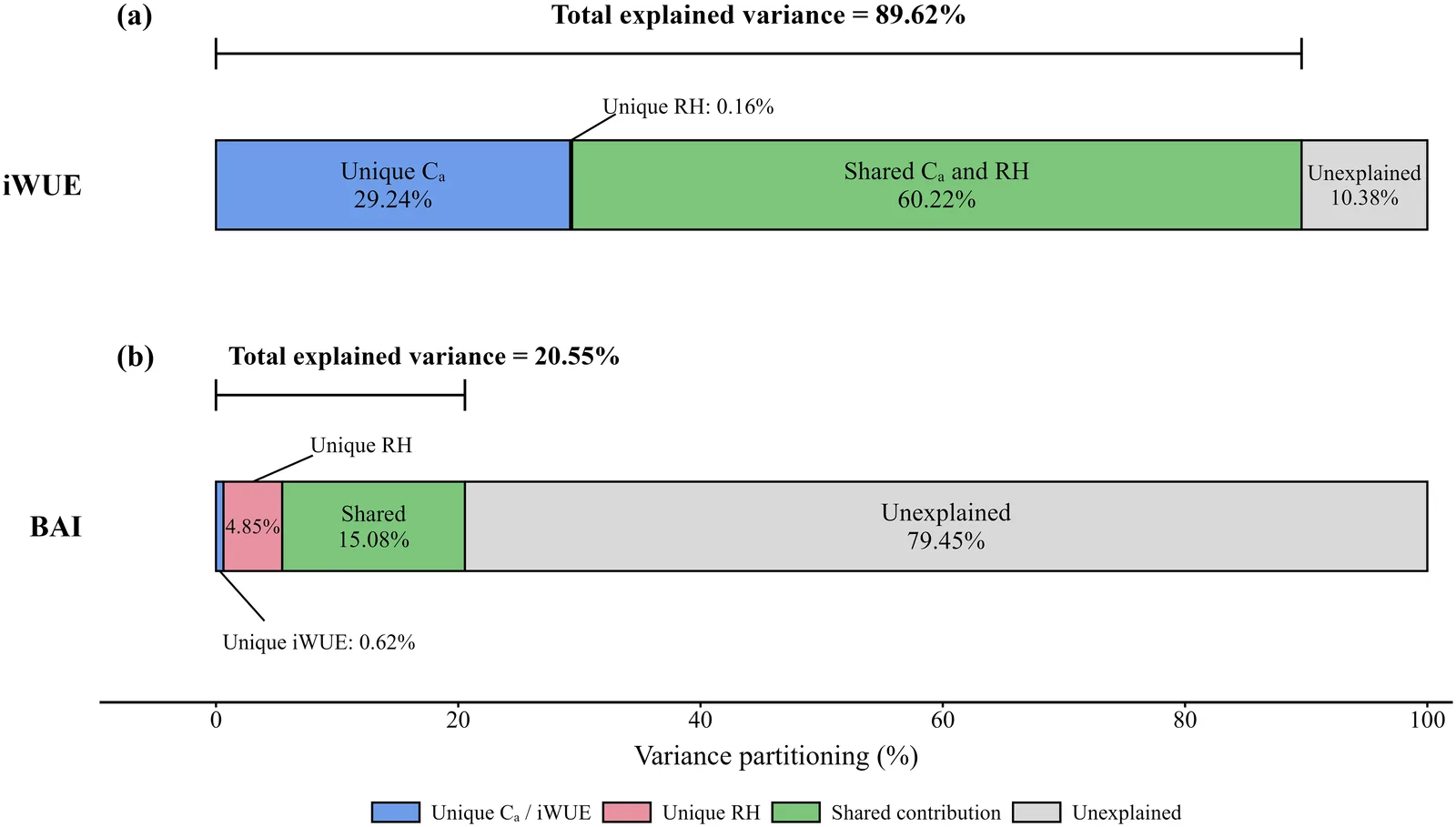
Results of commonality analysis among C_a_, RH, iWUE, and BAI: **(a)** variance partitioning of iWUE explained by C_a_ and RH; **(b)** variance partitioning of BAI explained by iWUE and RH.

Commonality analysis quantitatively partitioned the contributions of C_a_ and RH to iWUE, as well as those of iWUE and RH to BAI (**Figure 9**). C_a_ and RH jointly accounted for 89.62% of the variance in iWUE, with their shared contribution accounting for 60.22%, while C_a_ contributed 29.24% independently and RH contributed only 0.16% independently. The remaining 10.38% of the variance was unexplained. In terms of radial growth, iWUE and RH collectively explained 20.55% of the variance in BAI, with their shared contribution representing 15.08%, and unique contributions of 0.62% from iWUE and 4.85% from RH. The remaining 79.45% of the variance was unexplained.

## 4 Discussion

### 4.1 Variations of tree-ring carbon isotopes and iWUE

From 1964 to 2023, temperatures and VPD increased significantly, while RH, SPEI, and PDSI declined markedly, indicating intensified atmospheric drying and increasing regional moisture deficits. In this context, prior to 2012, δ¹³C showed a steady increase, whereas Δ¹³C continuously decreased. Within the framework of carbon isotope discrimination, this pattern is consistent with increasing stomatal restriction under progressively drier conditions. Reduced stomatal conductance limits the diffusion of atmospheric CO_2_ into leaves, causing C_i_ to increase more slowly than C_a_, thereby weakening carbon isotope discrimination, enriching tree-ring δ¹³C, and increasing iWUE (Mathias and Thomas, 2021). Similarly, long-term trends of δ¹³C in four tree species from the mountainous areas of Beijing also displayed an increasing pattern (Lu et al., 2024), which aligns with our findings. However, studies conducted in other regions observed a continuous decline in δ¹³C throughout their respective study periods (Li et al., 2021, 2024). These contrasting patterns suggest that long-term isotope responses depend on the relative influences of atmospheric CO_2_ enrichment, water availability, photosynthetic assimilation, and stomatal regulation, which may differ among species and climatic regions.

After approximately 2012, tree-ring δ¹³C decreased, whereas Δ¹³C and C_i_/C_a_ increased, indicating enhanced isotope discrimination and relatively greater internal CO_2_ availability (Wang et al., 2025a). This change coincided with generally higher annual precipitation and temporary increases in SPEI and PDSI, suggesting that a partial relaxation of water limitation may have contributed to the observed isotope shift. This temporary improvement in regional moisture conditions may have occurred within a broader context of concurrent changes in large-scale atmospheric circulation. In 2012, the East Asian summer monsoon was stronger than normal, while the relatively northward position of the western North Pacific subtropical high favored enhanced transport of warm and moist air toward North China and the Taihang Mountains (Zhao et al., 2015). Meanwhile, ENSO conditions in the tropical Pacific underwent a noticeable phase adjustment, whereas the North Pacific PDO remained predominantly in its negative phase (Wu et al., 2022; Sun et al., 2021). These basin-scale modes of Pacific climate variability may have further modulated the East Asian summer monsoon, the western North Pacific subtropical high, moisture transport, and regional precipitation patterns. This episodic increase in moisture may have mitigated the previously intensified stomatal constraints, resulting in relatively higher stomatal conductance, a rise in C_i_/C_a_, and greater isotopic fractionation (Brennan et al., 2025). Accordingly, the shift around 2012 is more likely to reflect a phase transition in carbon–water regulation than a single-year anomaly, associated with the combined effects of regional moisture recovery and large-scale circulation adjustments, indicating a change in the dominant controls over C_i_/C_a_ between the pre- and post-2012 periods.

Changes in photosynthetic capacity may also have contributed to the increase in C_i_/C_a_ after 2012. Elevated temperatures or drought stress can constrain photosynthetic assimilation, thereby reducing CO_2_ consumption and resulting in relatively higher C_i_. This increase in C_i_ subsequently promotes an increase in C_i_/C_a_ and enhances isotopic fractionation (Wang et al., 2025b). The post-2012 increase in C_i_/C_a_ was accompanied by a shift in observed iWUE from approximating the “C_i_ constant” scenario in the early period to falling between the “C_i_ constant” and “C_i_/C_a_ constant” scenarios in the later period, indicating that the long-term increase in iWUE is driven by both the continuous rise in atmospheric CO_2_ and dynamic adjustments in stomatal regulation (Pu and Lyu, 2023). However, the positive effect of rising C_a_ on tree growth has proven insufficient to mitigate the negative impacts of temperature-induced water stress (Poulter et al., 2013). Under such conditions, conservative stomatal regulation may enhance water conservation and iWUE, while simultaneously limiting CO_2_ uptake and the growth benefits associated with CO_2_ fertilization.

Overall, under the combined effects of sustained warming and rising atmospheric CO_2_, *Larix principis-rupprechtii* adjusted its carbon–water relations through changes in the C_i_/C_a_ ratio, exhibiting clear stagewise response patterns and physiological plasticity. Within this framework, the iWUE of *Larix principis-rupprechtii* showed a significant increasing trend over the study period, consistent with the response patterns observed in *Pinus tabuliformis* in northern China (Li et al., 2023). Analyses of extensive tree-ring δ¹³C datasets indicate that global tree iWUE has increased by approximately 40% since 1901 (Mathias and Thomas, 2021), suggesting that rising atmospheric CO_2_ represents a key driver of long-term iWUE enhancement. However, the magnitude of this increase varies among vegetation types and climatic regions. In cold and arid regions, such as the semi-arid areas of northwestern China, increases in tree iWUE can exceed 50%, whereas in warm and humid tropical and subtropical forests, the increases are lower, typically ranging from 25% to 35% (Rahman et al., 2019). Across different vegetation types, cumulative iWUE increases are generally greater in coniferous forests (30%–55%) than in broadleaf forests (25%–40%) (Mathias and Thomas, 2021). In this study, the iWUE of *Larix principis-rupprechtii* increased by approximately 51.96% from 1964 to 2023, corresponding to an annual increment of about 0.9 μmol mol^-^¹ a^-^¹. This increase exceeds the global average and falls within the upper range of global coniferous forest iWUE enhancement, suggesting that, in addition to the effects of CO_2_ background, regional water limitations and atmospheric drying may have contributed to the relatively large increase in iWUE. In northern China, *Platycladus orientalis* exhibited a linear iWUE increase of up to 0.88 μmol mol^-^¹ a^-^¹ over a comparable study period (Lu et al., 2018), closely matching the magnitude observed in this study. These findings suggest that sustained atmospheric drying in the central-northern Taihang Mountains may have promoted more conservative stomatal regulation, thereby increasing iWUE and reducing transpiration water loss. Nonetheless, the relative contributions of stomatal versus assimilation limitations during the 2012 turning point remain unclear. Because iWUE was derived solely from tree-ring δ¹³C, changes in stomatal conductance and photosynthetic assimilation cannot be independently distinguished. Therefore, the inferred stomatal responses should be regarded as plausible physiological interpretations rather than direct evidence. A more robust distinction between stomatal and photosynthetic controls requires the combined analysis of tree-ring δ¹³C and δ¹^8^O. Future research should integrate this dual-isotope approach with higher-resolution observations of moisture and atmospheric dryness, such as soil moisture and vapor pressure deficit, as well as leaf gas exchange and stem sap flow, to more precisely identify the relative roles of the processes underlying C_i_/C_a_ variability.

### 4.2 Climate responses of Δ¹³C

Monthly-scale correlation analysis indicated that Δ¹³C exhibited pronounced seasonal sensitivity to moisture conditions. Overall, Δ¹³C was positively correlated with RH, precipitation, SPEI and PDSI, suggesting that tree-ring carbon isotope fractionation in the study area was closely associated with seasonal water availability. Relatively wet conditions promote higher stomatal conductance, which increases leaf-internal CO_2_ concentration and enhances isotopic fractionation (Zeng et al., 2024). Moreover, significant correlations between Δ¹³C and moisture variables in October of the previous year suggest that antecedent water conditions may exert lagged effects on physiological processes during the following growing season. Favorable autumn moisture conditions may improve pre-winter physiological status and soil-water recharge, while also affecting the formation and subsequent use of stored non-structural carbon during early growth in the following year (Castagneri et al., 2018; Namvar et al., 2024). The significant negative correlations between Δ¹³C and VPD in September and October of the previous year further support the potential carry-over effects of autumn atmospheric dryness. These findings are consistent with tree-ring isotope studies in the Tianmu Mountains (Liu et al., 2018) and the upper Tarim River (Ye et al., 2023), underscoring the dominant influence of moisture factors on isotopic signals.

Δ¹³C exhibits a significant negative correlation with both VPD and temperature variables, indicating that increased atmospheric evaporative demand and intensified water stress reduced carbon isotope discrimination. Under such conditions, enhanced stomatal closure reduces C_i_/C_a_, weakens carbon isotope discrimination, and ultimately lowers Δ¹³C (Kelly et al., 2016). The concurrent negative responses to temperature and VPD further indicate that the effect of warming on isotope discrimination was mediated through increased atmospheric moisture demand. Consequently, C_i_/C_a_ may become increasingly sensitive to regional aridification and its seasonal variability (Keen et al., 2022). Similarly, studies conducted in the Loess Plateau (Ren et al., 2022) have identified significant correlations between Δ¹³C and summer temperature, accompanied by inverse relationships with precipitation and RH. These findings indicate that summer heat and atmospheric drying can intensify the effects of water limitation. Additionally, the significant negative correlation between Δ¹³C and autumn temperatures of the previous year indicates that elevated autumn temperatures may increase evaporative demand and exacerbate water stress, thereby affecting stomatal regulation, reducing isotopic fractionation, and transmitting these effects to the following growing season through the reutilization of non-structural carbon (Namvar et al., 2024). At the single-month scale, the strongest relationship was observed between Δ¹³C and August VPD, whereas the combined-month analysis identified April–August VPD as the variable most strongly associated with Δ¹³C. These results indicate that atmospheric evaporative demand during the main growing season was particularly important for carbon isotope discrimination. Taken together, seasonal water availability appears to determine the general direction of Δ¹³C variation, while warming and elevated VPD may strengthen water limitation by increasing atmospheric evaporative demand.

### 4.3 Climate responses of iWUE and tree growth and their coupling relationship

Annual climate correlations indicate that iWUE is significantly positively correlated with annual mean temperature and VPD, and significantly negatively correlated with annual mean RH, PDSI and SPEI. In contrast, the correlation between iWUE and precipitation is weak and nonsignificant. These relationships are consistent with enhanced physiological regulation under warming and drying conditions, in which increased atmospheric evaporative demand and moisture deficits may promote reductions in stomatal aperture and conductance. This reduction limits the supply of leaf-internal CO_2_, decreases the C_i_/C_a_ ratio, and consequently enhances iWUE (Mathias and Thomas, 2021). Moreover, VPD directly represents atmospheric evaporative demand, whereas RH, SPEI, and PDSI capture atmospheric moisture conditions and broader climatic water deficits (Yuan et al., 2025). Collectively, these indicators provide a more comprehensive assessment of drought stress than annual precipitation alone, which may account for their stronger and more consistent associations with iWUE. In contrast, BAI exhibits no significant correlations with annual mean temperature, precipitation, SPEI, PDSI, or VPD, and is only weakly yet significantly negatively correlated with annual mean RH, indicating that radial growth demonstrates limited direct linear responses to interannual climate variations. Additionally, the original iWUE and BAI series were significantly positively correlated, whereas this relationship became nonsignificant after first-order differencing. This contrast indicates that the apparent association between iWUE and BAI was mainly attributable to shared long-term trends rather than consistent year-to-year coupling. The 11-year moving correlations further showed that the relationship varied through time, supporting a temporally nonstationary coupling between physiological water-use regulation and radial growth. Similar decoupling between long-term increases in iWUE and interannual growth variation has been reported in northwestern China and other water-limited forests (Liu et al., 2014a; Chen et al., 2022).

Commonality analysis showed that iWUE and RH together account for 20.55% of the variation in BAI, with their shared contribution comprising 15.08% and the independent contribution of iWUE only 0.62%. This suggests that the influence of iWUE on radial growth is predominantly modulated by water conditions and exhibits significant scenario dependency. This finding is consistent with studies conducted on *Larix gmelinii* and *Pinus sylvestris* var. *mongolica* in the Greater Khingan Mountains (Zhang et al., 2018; Chen et al., 2022). The combined effects of iWUE and climatic variables accounted for only a limited proportion of the variation in radial growth, while the independent contribution of iWUE remained relatively minor. When water supply is sufficient, increases in iWUE are more likely to provide growth advantages. However, under sustained water stress in the study area, elevated evaporative demand due to warming and soil water deficits constrain the allocation of photosynthates to radial growth, preventing increases in iWUE from fully translating into gains in BAI (Olano et al., 2014). Moreover, the variability in iWUE was strongly associated with rising atmospheric CO_2_ and moisture conditions, whereas BAI exhibits significantly lower explainable variation, indicating that radial growth did not respond synchronously to increases in iWUE. This suggests that iWUE more directly reflects physiological carbon–water regulation under water-limited conditions, whereas radial growth integrates a broader range of climatic, physiological, and site-related controls.

The use of whole-ring cellulose may obscure seasonal differences between earlywood and latewood, which are formed at different stages of the growing season and may differ in their dependence on stored and newly assimilated carbon (Castagneri et al., 2018). Consequently, whole-ring δ¹³C-derived iWUE may not fully correspond to the seasonal processes controlling annual radial growth, thereby weakening its apparent relationship with BAI. In addition, higher iWUE under drought does not necessarily indicate greater total carbon assimilation, because stomatal closure may increase iWUE while simultaneously restricting CO_2_ uptake. Available assimilates may also be preferentially allocated to maintenance respiration, non-structural carbohydrate storage, root growth, osmotic adjustment, defense, and hydraulic maintenance or repair rather than to cambial activity and wood formation. These seasonal differences and competing carbon demands may diminish the direct translation of climate signals and changes in iWUE into radial growth responses (Penuelas et al., 2011; Fernández‐de‐Uña et al., 2016). Overall, the observed increase in iWUE without concurrent growth enhancement is consistent with global reports (Rahman et al., 2019; Wang et al., 2021; Zhang et al., 2026), indicating that the potential physiological benefits of rising CO_2_ may be partly offset by atmospheric drying, water limitation, and competing carbon demands under changing environmental conditions.

### 4.4 Forest adaptation strategies and future prospects under climate change in the Central-Northern Taihang Mountains

Over the past 60 years, the Taihang Mountains have experienced sustained warming accompanied by intensified atmospheric drying. *Larix principis-rupprechtii* exhibits conservative stomatal regulation and water-use strategies by reducing C_i_/C_a_ and enhancing iWUE. However, the long-term increases in iWUE have not consistently resulted in synchronous increases in BAI, and growth variability is predominantly driven by water availability, indicating that projecting future enhancements in carbon sinks based solely on increases in iWUE may be misleading. Forest management strategies should account for the specific environmental conditions of the central-northern Taihang Mountains. Shallow and spatially heterogeneous soils, steep slopes, pronounced seasonal water limitation, and highly concentrated monsoon precipitation may reduce soil-water storage capacity while increasing surface runoff and erosion risks (Del Campo et al., 2022). Under these conditions, stand-density regulation and structural optimization should aim to reduce excessive competition for water while avoiding overly intensive canopy opening, which may enhance soil evaporation and erosion. Moderate thinning, retention of understory vegetation and litter layers, and measures that promote rainfall infiltration and reduce surface runoff may help improve soil-water conservation (Keenan, 2015). In addition, selecting drought-tolerant provenances with stronger rooting capacity, greater hydraulic safety, and better adaptation to seasonal water deficits may contribute to maintaining stand stability under continued warming and drying (Liang et al., 2025). The present study provides site-based evidence from a single high-elevation stand and does not encompass broader stand-density or environmental gradients. In addition, without continuous site-level soil moisture observations, the relative effects of atmospheric dryness and soil water limitation, as well as their lagged influences on tree physiology and radial growth, remain to be further distinguished. Future studies should include replicated stands across gradients of density, elevation, slope aspect, and soil depth, together with continuous observations of soil moisture, leaf gas exchange, stem sap flow, and cambial activity. Event-based analyses focusing on extreme drought, post-drought recovery, and successive drought years would further clarify resistance, resilience, and drought legacy effects. Combining tree-ring δ¹³C and δ¹^8^O, separate earlywood and latewood analyses, and process-based models such as 3-PG would also improve predictions of carbon–water coupling and carbon-sink stability in the central-northern Taihang Mountains (Wang et al., 2025c). Such integrated observations and modeling would provide a stronger basis for translating tree-ring physiological evidence into adaptive forest-management practices.

## 5 Conclusion

This research elucidates the mechanisms governing the responses of tree-ring stable carbon isotopes and radial growth in *Larix principis-rupprechtii* to climate change in the central-northern Taihang Mountains. The δ¹³C and Δ¹³C values displayed notable inter-annual variability and a stepwise shift around 2012, indicating adjustments in the relative limitations imposed by stomatal regulation and photosynthetic processes. With continued atmospheric CO_2_ increase, the rise in C_i_ was smaller than that of C_a_, resulting in an overall decline in C_i_/C_a_ ratio and a significant 51.96% augmentation in iWUE. These results indicate that both the background effects of CO_2_ and water stress collaboratively propel the long-term improvement in iWUE, reflecting an adaptive strategy where trees optimize stomatal behavior to enhance water-use efficiency under water-limited circumstances. Δ¹³C was highly sensitive to moisture levels during the growing season, exhibiting a significant positive correlation with relative humidity and a significant negative correlation with VPD, underscoring water availability as a key determinant of carbon isotope discrimination. Rising temperatures exacerbate water stress by increasing evapotranspiration, which may further reduce isotopic discrimination. In comparison, iWUE responded more strongly to climatic variables than BAI, whereas BAI exhibited a significant relationship only with RH. Although the long-term trends of iWUE and BAI were consistent, their inter-annual coupling was weak. Commonality analysis indicated that the independent contribution of iWUE to growth was limited, with its effects primarily mediated by synergistic interactions with water availability. This finding suggests that increased iWUE does not necessarily translate into sustained growth gains, as the effects of CO_2_ fertilization may be offset by intensified atmospheric drying and water deficits. Overall, *Larix principis-rupprechtii* in the central-northern Taihang Mountains enhances iWUE through conservative stomatal strategies to cope with warming and drying pressures. However, the optimization of carbon–water coupling is expressed more through physiological regulation than through proportional increases in wood production. Future studies should integrate high-resolution moisture and atmospheric dryness indices with multi-isotope physiological observations to guide adaptive management and assess carbon sinks in mountain coniferous forests.

## Data Availability

The original contributions presented in the study are included in the article/**Supplementary Material**. Further inquiries can be directed to the corresponding authors.
