# Supplementary material for: Tree-ring δ¹³C and growth responses of Larix principis-rupprechtii to climate change in the Central-Northern Taihang Mountains
Source: Front Plant Sci. 2026 Aug 13;17:1896087. doi: 10.3389/fpls.2026.1896087 (PMC13518205; doi:10.3389/fpls.2026.1896087)
Supplement: Supplementary file 1 [file SupplementaryFile1.docx]

Supplementary Information

Supplementary Fig. S1. Temporal variations in the standardized tree-ring-width chronology (STD, red line), basal area increment (BAI, blue line), and sample depth (gray line) from 1918 to 2023. The STD chronology was significantly positively correlated with BAI (r=0.59, *p*<0.001, n=106).
